# Unsupervised super-resolution reconstruction of hyperspectral histology images for whole-slide imaging

**DOI:** 10.1117/1.JBO.27.5.056502

**Published:** 2022-05-16

**Authors:** Ling Ma, Armand Rathgeb, Hasan Mubarak, Minh Tran, Baowei Fei

**Affiliations:** a University of Texas at Dallas, Department of Bioengineering, Richardson, Texas, United States; b Tianjin University, State Key Laboratory of Precision Measurement Technology and Instruments, Tianjin, China; c University of Texas at Dallas, Center for Imaging and Surgical Innovation, Richardson, Texas, United States; d University of Texas Southwestern Medical Center, Department of Radiology, Dallas, Texas, United States

**Keywords:** hyperspectral imaging, super-resolution reconstruction, unsupervised, RGB guidance, image classification

## Abstract

**Significance:**

Hyperspectral imaging (HSI) provides rich spectral information for improved histopathological cancer detection. However, acquiring high-resolution HSI data for whole-slide imaging (WSI) can be time-consuming and requires a huge amount of storage space.

**Aim:**

WSI using a color camera can be achieved with fast speed, high image resolution, and excellent image quality due to the established techniques. We aim to develop an RGB-guided unsupervised hyperspectral super-resolution reconstruction method that is hypothesized to improve image quality while maintaining the spectral characteristics.

**Approach:**

High-resolution hyperspectral images of 32 histologic slides were obtained via automated WSI. High-resolution RGB histology images were registered to the hyperspectral images for RGB guidance. An unsupervised super-resolution network was trained to take the downsampled low-resolution hyperspectral patches (LR-HSI) and high-resolution RGB patches (HR-RGB) as inputs to reconstruct high-resolution hyperspectral patches (HR-HSI). Then, an Inception-based network was trained with the HR-RGB, original HR-HSI, and generated HR-HSI, respectively, for whole-slide histopathological cancer detection.

**Results:**

Our super-resolution reconstruction network generated high-resolution hyperspectral images with well-maintained spectral characteristics and improved image quality. Image classification using the original hyperspectral data outperformed RGB because of the extra spectral information. The generated hyperspectral image patches further improved the results.

**Conclusions:**

The proposed method potentially reduces image acquisition time, saves storage space without compromising image quality, and improves the image classification performance.

## 1 Introduction

Computer-aided pathology (CAP) is an active research area that aims to improve the reproducibility and objectivity of pathological diagnosis and save time in routine examination.^1^ Machine learning techniques, particularly deep learning models, have played an essential role in CAP.^2^ Various studies of deep learning-based cancer detection in whole-slide digitized histology images have been investigated, and most of them were carried out in RGB images. Hyperspectral imaging (HSI) is a noncontact and label-free imaging modality that has emerged in the medical imaging field. It captures the spatial and spectral information of the imaged tissue, revealing the chemical composition and morphological features in a single image modality and thus offering more fine features that are potentially useful for image segmentation and classification. Studies have proven the usefulness of HSI in microscopy applications.^3^^–^^15^ One of the promising applications for this technology is employing HSI for whole-slide imaging (WSI) to aid the histopathological cancer detection of tissue samples because HSI not only provides a reproducible and quantitative diagnosis of the slides but also improves the classification results compared with RGB.^3^^,^^5^ Although various steps such as fixation and embedding during the preparation of the histological samples may alter a few tissue features, such as the texture and the water content, lots of important molecules such as proteins are preserved in the tissue slides. The light absorption, scattering, and autofluorescence of these molecular components all contribute to the spectral characteristics in hyperspectral histologic images,^6^^,^^16^ which provide a different perspective from the traditional gross-level reflectance HSI. Even for the dyes, HSI can expand the three-channel color information into a much wider spectral dimension, which might increase the discriminability. Nakaya et al.^17^ used a support vector machine classifier and the average spectra of nuclei extracted from hyperspectral images for colon cancer detection. Ishikawa et al.^18^ proposed a pattern recognition method named hyperspectral analysis of histopathological slides based on stain spectrum to process the spectra of the histologic hyperspectral images for pancreatic tumor nuclei detection. Ortega et al.^3^ implemented automatic breast cancer cell detection in hyperspectral histologic images with an average testing AUC of 0.90. The comparison between the classification results using HSI and RGB suggests that HSI outperforms RGB. Our previous studies investigated the feasibility and usefulness of HSI for head and neck squamous cell carcinoma (SCC) nuclei detection in histologic slides.^4^^,^^19^ The comparison between HSI, HSI-synthesized RGB, and RGB indicates that the extra spectral information from HSI can improve the outcome. We also investigated whole-image histopathological cancer detection and proved the advantage of using HSI for head and neck cancers.^5^^,^^20^

**Fig. 1:**
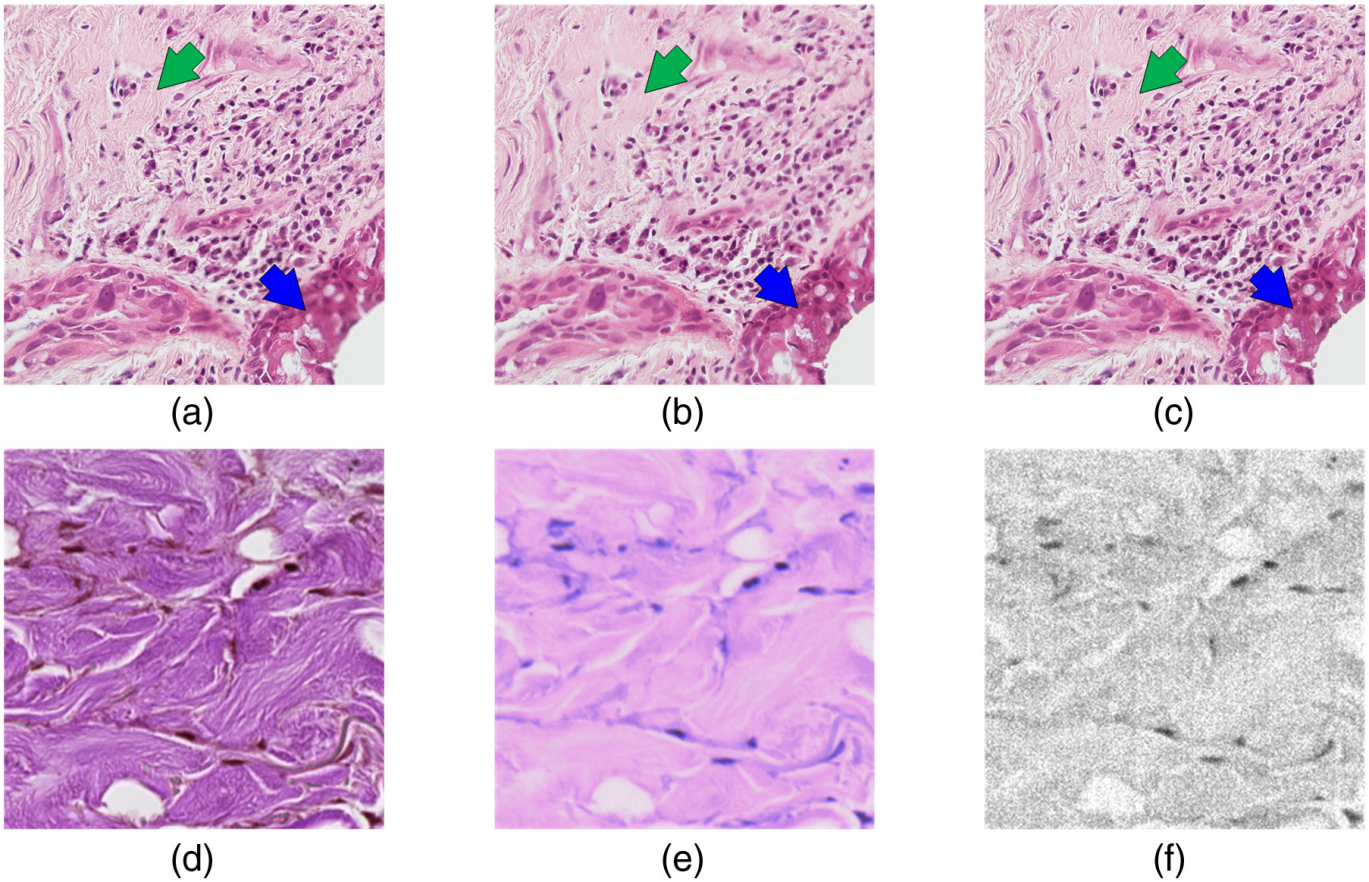
Incidences of low image quality of hyperspectral images. (a) and (b) Two images showing how tissues with different thicknesses in the same image cannot be in focus at the same time. (c) RGB histology image acquired with EFI function integrated with the state-of-the-art microscope. (d) High-resolution digital histology image of a thyroid slide with high image quality. (e) HSI-synthesized RGB image of the same region as (d) but with lower contrast because of the unsatisfying focusing during the slide scanning without autofocusing. (f) The first band from the hyperspectral image of the same region as (d) with a lot of noise due to low sensitivity.

There are a few difficulties when applying hyperspectral microscopy in real clinical situations. First, the size of a hyperspectral image file is large. For example, one hyperspectral image with a dimension of 3600 pixels × 2048 pixels × 150 bands saved in single precision requires about 4 GB of storage space. A whole-slide hyperspectral image acquired at a low magnification easily exceeds 100 GB. In some applications such as hematology, high magnification is necessary, which may put an extreme burden on data storage devices. Current limitations on data storage makes it difficult to establish a comprehensive hyperspectral microscopic dataset, which is essential for a thorough study of deep learning methods. Second, the acquisition of hyperspectral images can be time-consuming. Most hyperspectral microscopy studies used push-broom or spectral-scanning systems to obtain a sufficient spectral and spatial resolution. The scanning process of each image can take several seconds, which greatly increases the acquisition time of a whole-slide image. Even though a snapshot hyperspectral camera speeds up the acquisition, its intrinsic low spatial resolution would not meet the requirement of some applications in which fine structures in the slides need to be seen. Third, it is more difficult to implement autofocusing with a hyperspectral camera than a color camera. During the scanning of the histological slides, the change of slide thickness can result in unfocused images, and cellular components from different layers can become blurry. State-of-the-art microscopes and whole-slide scanners are integrated with autofocusing and extended focal imaging (EFI) algorithms to solve these problems; they are based on the acquisition of a stack of RGB images along the Z axis. For example, in Figs. 1(a) and (b), the green and blue arrows point at two regions where tissues with different thicknesses cannot be in focus simultaneously. With the EFI algorithm, a stack of images is acquired at different Z positions and then flattened to make an evenly focused image, as shown in Fig. 1(c). Although it is possible to apply such image enhancement algorithms to HSI, it would further prolong the scanning time tremendously. Finally, some bands in a hyperspectral image, especially the first and last several bands, can have large amounts of noise due to the low sensitivity. Both the unsatisfied focus and noise can result in low image quality and missing spatial details, i.e., high-frequency information, as shown in Figs. 1(d)–1(f). On the other hand, whole-slide scanning with a color camera has become routine in the field of pathology. Digital histology images that are acquired with high-quality color cameras have very high image resolution and contrast. Making use of such advantageous features with HSI potentially improves the image quality of hyperspectral images.

Hyperspectral image super-resolution reconstruction,^21^^–^^23^ also known as HSI pansharpening^24^ or HSI spatial resolution enhancement,^25^ is a technique that uses the spatial information from a high-resolution panchromatic image (PAN) and the color information from a low-resolution spectral image to generate a high-resolution spectral image. Different from the concept of “super-resolution imaging” that aims to increase the optical resolution of an imaging system, super-resolution image reconstruction uses various digital image processing techniques to generate images with higher resolutions than the original ones. It has been developed mainly for remote sensing applications. Previously proposed HSI super-resolution reconstruction methods, such as component substitution, multiresolution analysis, hybrid methods, and model-based methods, either result in spectral distortion, generate blurry results, or add complexity to implementation.^24^^,^^26^ In recent years, deep learning algorithms have been explored for the super-resolution reconstruction of hyperspectral images. Masi et al.^27^ adopted a three-layer convolutional neural network (CNN) named PNN to reconstruct high-resolution multispectral images from the stack of interpolated low-resolution multispectral images and high-resolution PAN. However, the network had a limited learning ability due to the shallow architecture. Yang et al.^28^ proposed a ResNet-based architecture named PanNet that did not fully exploit the spatial information due to the use of the high-pass filter. Yao et al.^29^ implemented pixelwise regression for hyperspectral pansharpening using a U-Net. Zheng et al.^26^ developed a two-part framework that first enhanced the spatial resolution in hyperspectral images through contrast limited adaptive histogram equalization and then used a deep residual neural network to further boost the fusion accuracy.

Since many microscopes and whole-slide scanners can have more than one camera that share the same field of view (FOV), applying the super-resolution reconstruction technique for hyperspectral microscopy and WSI is feasible. Considering the easy acquisition and wide utilization of high-quality RGB histology images, we propose using high-resolution RGB images to guide the super-resolution reconstruction of high-resolution hyperspectral images. Dey et al.^30^ adopted a linear degradation model to super-resolve imaging mass spectrometry with hematoxylin and eosin (H&E)-stained histology images, which neglected the nonlinearity in spectral responses and resulted in slightly noisy images. Jiang et al.^25^ used the matrix factorization method to fuse high-resolution RGB histology images and low-resolution hyperspectral microscopic images acquired with a push-broom camera, but the method was only tested for spatial enhancement scales of 3× and 4×. Moreover, none of these studies have investigated deep learning algorithms. Despite a wide variety of models proposed for super-resolution reconstruction in remote sensing, most of them were based on supervised learning methods.^26^^–^^29^^,^^31^^,^^32^ Using this technique, the reconstructed high-resolution hyperspectral image might inherit the noise from the reference hyperspectral image. In addition, the goal of many previous studies was to reconstruct images as close as possible to the real high-resolution spectral images, but what if we can generate images with improved image quality?

Ideally, the super-resolution reconstruction of hyperspectral microscopic images should take full advantage of the superb image quality of digital histology images while maintaining the spectral features that are critical for image classification. Therefore, in this work, we develop a simple yet effective unsupervised super-resolution reconstruction network that fuses the spatial information from the high-resolution RGB images and the spectral information from the low-resolution hyperspectral images. With the proposed method, it is possible to save the acquisition time and storage space for hyperspectral images, as well as compensate for the low quality of some bands in the hyperspectral images, thus promoting the application of HSI for WSI and automatic histopathological cancer detection.

## 2 Methods

### 2.1 Histologic Slides and Hyperspectral Data Acquisition

In this work, we utilized 32 H&E-stained histologic slides from 16 patients with head and neck SCC.^33^^,^^34^ Each slide was from either tumor (T) or normal (N) tissue, as confirmed and annotated by board-certified pathologists. The tissue was resected during a routine surgery, after which the specimen was inked, formalin-fixed, and paraffin-embedded. The top section of each specimen was obtained using a microtome and stained with H&E. High-resolution digital histology images of the slides were obtained using a whole-slide scanner with a 40× objective lens right after the preparation of the slides.

**Fig. 2:**
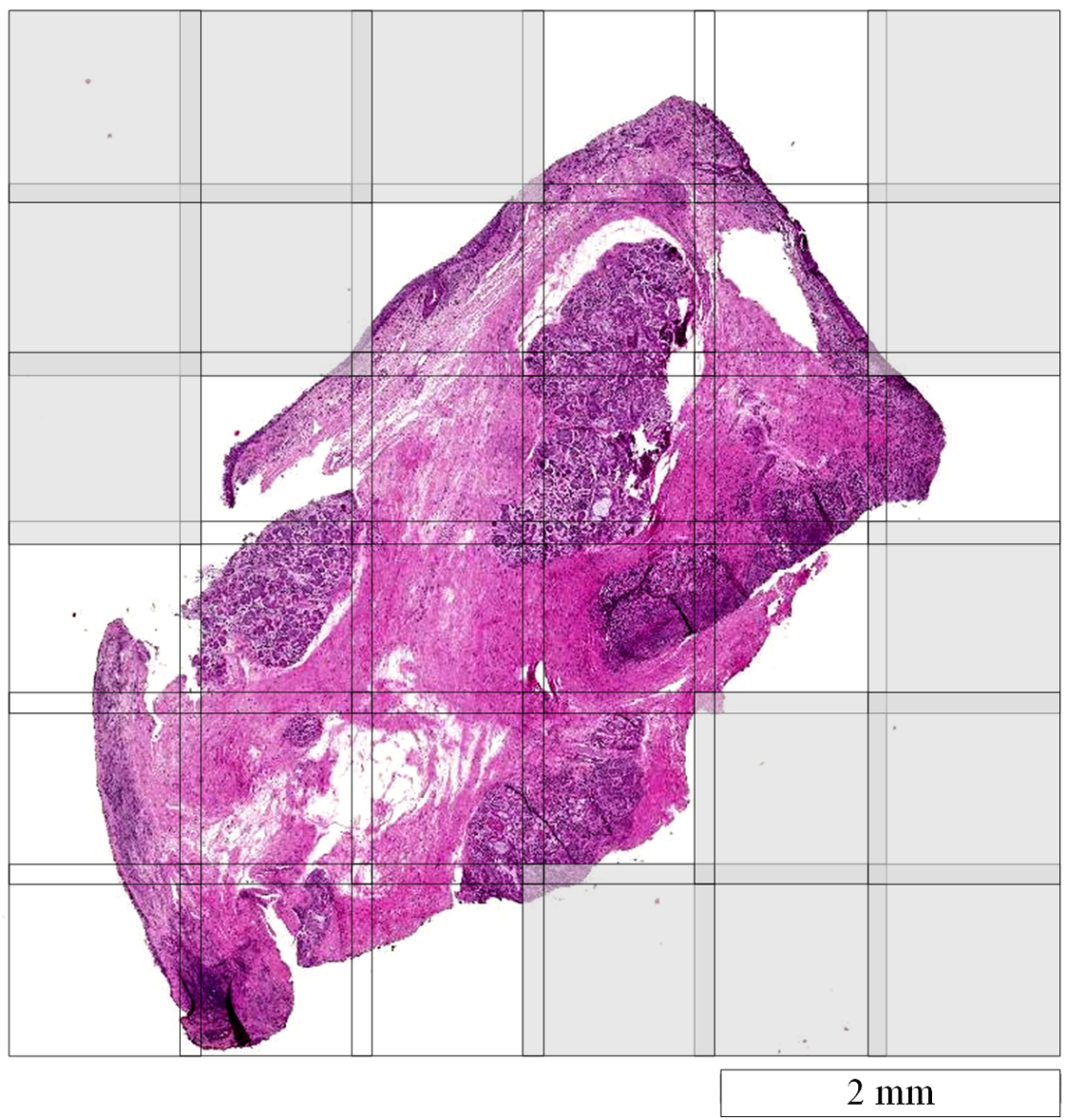
Whole-slide scanning with a step size of 1 mm. The gray grids are the positions where no image was acquired because minimal tissue was detected in the FOV.

For the acquisition of hyperspectral images, we used the hyperspectral microscopic imaging system that has been reported in our previous works^4^^,^^5^^,^^20^ to scan the slides. The dimensions of the hyperspectral images were 2000  pixels×2000  pixels×87  bands, covering a wavelength range of 470 to 720 nm. With the objective lens of 10× magnification, the FOV of the hyperspectral camera was about 1113  μm×1113  μm. A motorized stage was automated to move the slide along the horizontal and vertical directions with a step size of 1 mm, and one image was acquired at each step. The two adjacent images had an overlap area of about 113  μm wide (203 pixels). During the automatic scanning of the slide, the hyperspectral camera always waited for a few seconds after the stage motion to start image acquistion, giving us adequate time to focus the camera by manually adjusting the fine focusing knob without interrupting the scanning. Figure 2 shows an illustration of the scanning of an entire histologic slide. Each square in the figure indicates one hyperspectral image with a spatial size of 2000×2000  pixels, and every two adjacent images overlap 203 pixels with a scanning step size of 1 mm.

### 2.2 Data Preprocessing

Before the scanning of each slide, a white reference hyperspectral image was obtained at a blank area on the slide, and a dark current image was acquired automatically by the camera with the camera shutter closed. Afterward, each raw hyperspectral image was calibrated with the white reference and dark current images to obtain the transmittance of the tissue:

$$\text{Transmittance}(\lambda )=\frac{I_{\text{Raw}}(\lambda )-I_{\text{Dark}}(\lambda )}{I_{\text{White}}(\lambda )-I_{\text{Dark}}(\lambda )},$$ (1)

where Transmittance(λ) is the wavelength-dependent transmittance; $I_{\text{raw}}(\lambda )$ is the intensity value for wavelength λ in the raw hyperspectral image; and $I_{\text{white}}(\lambda )$ and $I_{\text{dark}}(\lambda )$ are the intensity values for wavelength λ in the white reference and dark current images, respectively.

**Fig. 3:**
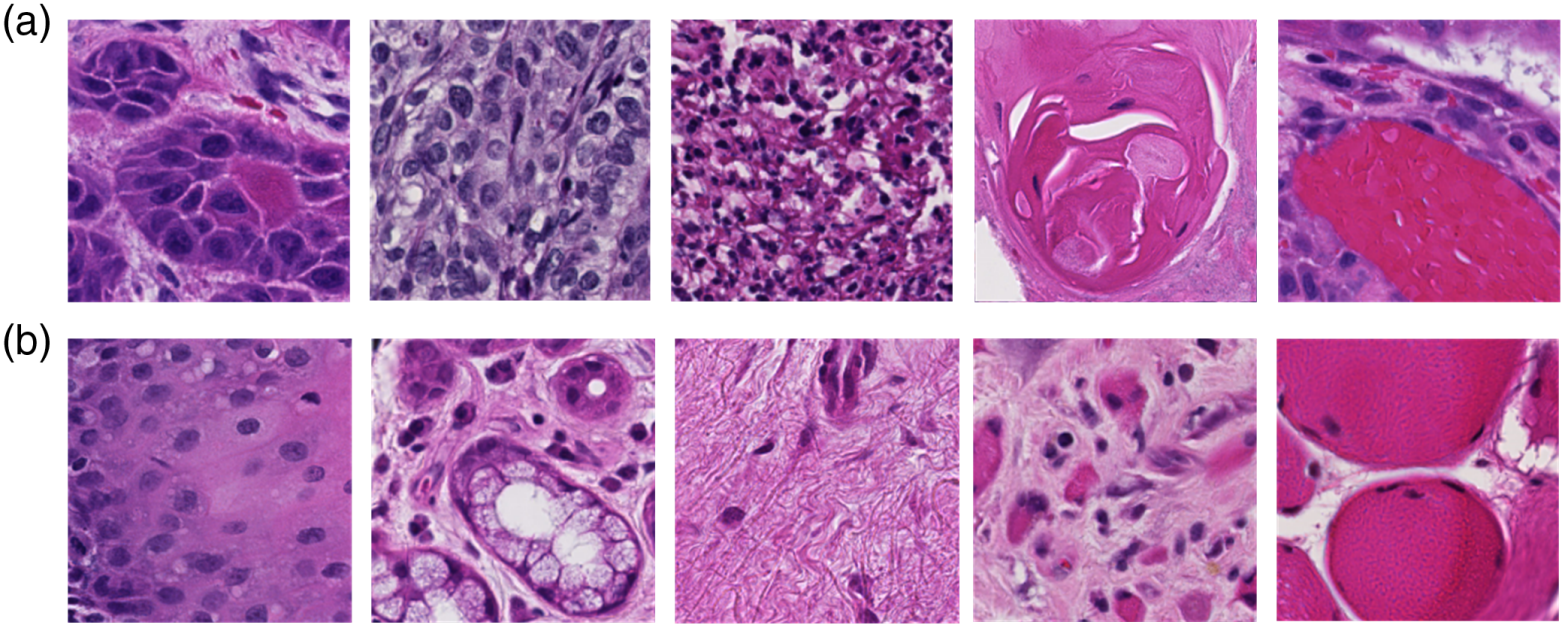
Coregistered RGB histological image patches (200×200  pixels) showing anatomical diversity. (a) Patches extracted from images of cancerous slides with various histological features, including nucleus atypia, basaloid SCC, SCC with chronic inflammation, SCC with keratin pearl, and SCC with hemorrhage. (b) Patches generated from images of normal slides with various histological features, including healthy stratified squamous epithelium, salivary glands, stroma, chronic inflammation, and skeletal muscle.

For each calibrated hyperspectral image, we synthesized an RGB image using a customized HSI-to-RGB transformation^5^ and a grayscale image by calculating the average of all 87 bands. The HSI-synthesized RGB images were generated for a better visualization of the FOV of hyperspectral images but not used for the training or validation of any network. The grayscale images were later used for image registration because registration algorithms cannot be directly applied on the 87-band hyperspectral image. The high-resolution RGB images that were used to guide the super-resolution reconstruction were cropped from digital histology images, which were originally imaged with a whole-slide scanner at 40× objective magnification, as stated in the previous section. We looked at each HSI-synthesized RGB image, found the corresponding region in the whole-slide digital histology image, and cropped the high-resolution RGB image with a slightly larger FOV than the HSI-synthesized RGB image. Then, all high-resolution RGB images were registered to their matching hyperspectral images (average grayscale images) using affine registration with the Oriented FAST and Rotated BRIEF (ORB) feature detector^35^ from the OpenCV package and were resized to a spatial size of 2000×2000  pixels. The registration achieved a pixel-to-pixel alignment between the high-resolution RGB images and the high-resolution hyperspectral images. Afterward, both the hyperspectral images and RGB images were cropped into 361 patches using a sliding window of 200×200  pixels with a step size of 100 pixels. Image patches with little tissue (<50% area of the whole patch) were removed from the dataset. The generated patches from hyperspectral images and coregistered RGB images are called high-resolution hyperspectral patches (HR-HSI) and high-resolution RGB patches (HR-RGB). Then, all HR-HSI were downsampled using a “box” (average) interpolation kernel by 2, 4, 5, 8, and 10 times, respectively, to generate low-resolution hyperspectral patches (LR-HSI) for five different super-resolution reconstruction networks. All image patches from the T and N slides were labeled either as “1” for tumor or “0” for normal according to the slide from which they were taken. Figure 3 shows some image patches with various anatomical structures from cancerous slides and normal slides, respectively. Table 1 shows the number of hyperspectral images and the number of image patches that we finally obtained for each slide.

**Table 1:** Summary of the hyperspectral histologic dataset.

Patient ID	Organ	Number of images			Number of patches		
		T slide	N slide	Total	Cancerous	Normal	Total
1	Larynx	58	42	100	16,444	9756	26,200
2	Hypopharynx	95	45	140	21,040	17,350	38,390
3	Larynx	38	62	100	11,077	16,924	28,001
4	Larynx	22	28	50	5015	6819	11,834
5	Larynx	58	23	81	14,526	5508	20,034
6	Larynx	29	10	39	6903	2139	9042
7	Larynx	28	16	44	7087	2992	10,079
8	Buccal mucosa	23	12	35	4888	1841	6729
9	Larynx	20	37	57	3979	8199	12,178
10	Larynx	13	19	32	3111	4604	7715
11	Larynx	53	24	77	13,573	3671	17,244
12	Larynx	23	17	40	5375	2694	8069
13	Larynx	58	28	86	16,318	6401	22,719
14	Larynx	46	28	74	12,519	6518	19,037
15	Larynx	35	17	52	8750	4238	12,988
16	Larynx	81	40	121	20,002	10,073	30,075
Total		680	448	1,128	170,607	109,727	280,334

**Fig. 4:**
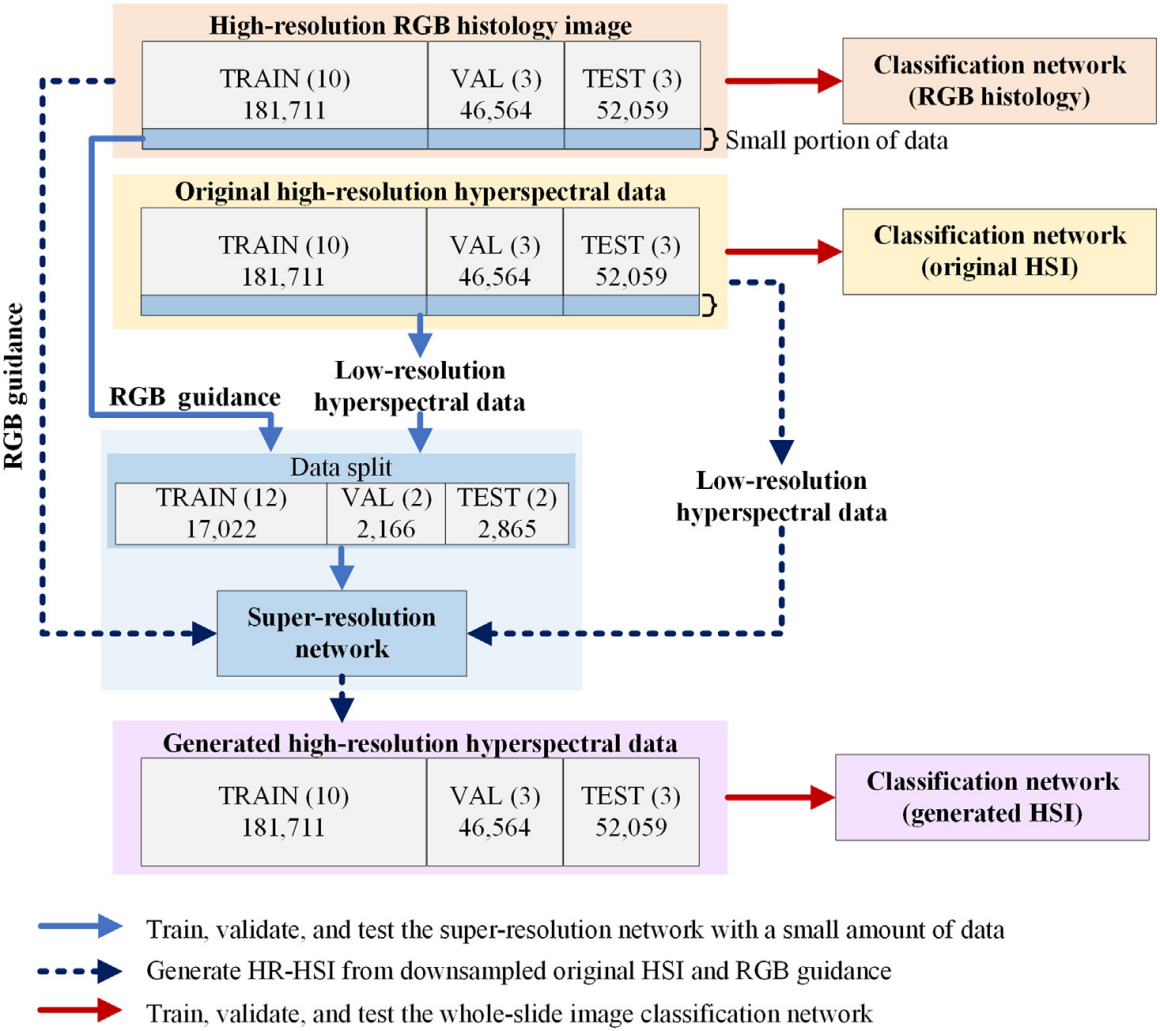
Illustration of experiment workflow and data partition. The numbers within the parentheses show how many patients were used for training (TRAIN), validation (VAL), and testing (TEST), with the numbers underneath showing the specific number of image patches used in each data group.

In this study, we first used a small portion of the entire dataset to train, validate, and test our proposed super-resolution reconstruction network. To make sure that the network is able to deal with the staining variation and thickness variation of different slides, which can cause the change of spectral signatures, we selected two images with minimal blank areas for each slide and used them as the “super-resolution dataset” instead of using lots of images from only one patient. In total, 22,053 patches from 64 images of 16 patients were selected, with 17,022 patches from 48 images of 12 patients used for training, 2166 patches from four images of two patients (#3 and #14) used for validation, and 2865 patches from four images of the rest two patients (#5 and #15) used for testing. Then, after the super-resolution reconstruction network was trained, high-resolution hyperspectral image patches were generated for all data using downsampled hyperspectral patches with RGB guidance, and an Inception-based CNN was trained for whole-slide image classification using (1) high-resolution RGB histology image patches, (2) original high-resolution hyperspectral image patches, and (3) generated high-resolution hyperspectral image patches, respectively. The data partition for three types of data was exactly the same. In total 181,711 images patches from 10 patients were used for training, 46,564 image patches from three patients (#3, #4, and #8) were used for validation, and 52,059 image patches from another three patients (#5, #14, and #15) were used for testing. The data that were previously used as the “super-resolution dataset” were not excluded for the image classification network to generate a classification probability map of the whole slide. Nevertheless, those data took only a small portion (<9%) in the entire dataset; thus they did not cause obvious bias for the classification results. The overall workflow and data partition are as shown in Fig. 4. The numbers within the parentheses show how many patients were used for training (TRAIN), validation (VAL), and testing (TEST), and the numbers underneath indicate the specific number of image patches included in each data group.

### 2.3 Unsupervised Super-Resolution Reconstruction Network

We developed an unsupervised super-resolution reconstruction network that takes the LR-HSI and HR-RGB as inputs and generates HR-HSI as well as LR-HSI for outputs. To consider different acquisition methods of low-resolution hyperspectral images, we trained five networks with different super-resolution scales. The first scenario is to use the same camera but reduce the magnification of the video adapter (e.g., from 1× to 0.5×), which will result in a smaller image size and lower image resolution. For this case, we trained a 2× super-resolution reconstruction network. The second scenario is to use a camera that has a relatively lower spatial resolution, such as the snapshot hyperspectral camera. For this case, we trained a 4× and a 5× network because 4×4 and 5×5 are the two most common mosaic patterns of snapshot hyperspectral cameras. Two other networks, namely 8× and 10×, were also implemented to evaluate whether the proposed method has the ability for larger-scale spatial enhancement.

**Fig. 5:**
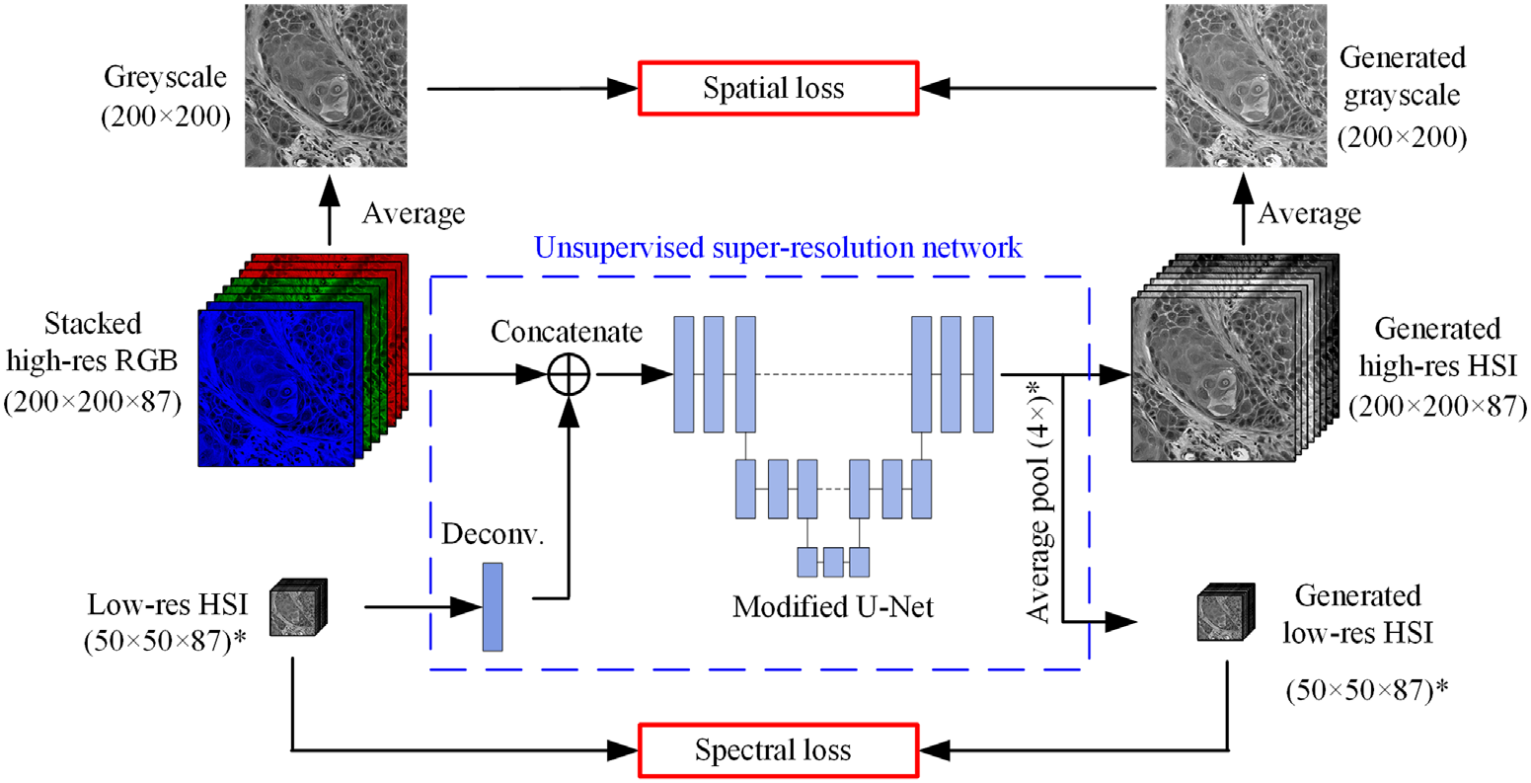
Architecture of the unsupervised 4× super-resolution reconstruction network, which takes stacked HR-RGB and LR-HSI as inputs and outputs HR-HSI and LR-HSI. Dimensions of the LR-HSI in the figure are for the 4× network only. For the 2×, 5×, 8×, and 10× networks, the dimensions of both the input and generated LR-HSI would be 100×100×87, 40×40×87, 25×25×87, and 20×20×87, respectively.^36^

Figure 5 shows the architecture of a 4× super-resolution reconstruction network as an example. The three channels (R, G, and B) of the input HR-RGB were duplicated by 35, 35, and 17 times, respectively, and stacked together to form an 87-band “stacked HR-RGB.” Specifically, the blue channel was duplicated less than other two channels because our hyperspectral camera did not cover the wavelength range of 370 to 470 nm, resulting in less blue-color related information in the hyperspectral images. The LR-HSI input (50×50×87) was first upsampled to 200×200×87 using a two-dimensional (2D) deconvolution layer and then concatenated with the stacked HR-RGB along the third dimension, i.e., the spectral dimension, to form a 200×200×174 patch. What followed is a modified U-Net architecture, which took the concatenated 174-band patch as input and output an HR-HSI patch. In addition, a 4×4 average pooling layer was applied to the generated HR-HSI to output an LR-HSI patch with the same size as the input LR-HSI. Note that the dimensions of both the input and generated LR-HSI in Fig. 5 are for a 4× network. For the 2×, 5×, 8×, and 10× networks, the dimensions would be 100×100×87, 40×40×87, 25×25×87, and 20×20×87, respectively. The details of the network are shown in Table 2.

**Table 2:** Super-resolution reconstruction network architecture.

Layer	Kernel/strides/padding	Output shape
Input_1: high-res RGB	Image input 1	200×200×87
Input_2: low-res HSI	Image input 2	W×W×87
Conv2D_Transpose	(S+1)×(S+1), S, ‘same’	200×200×87
Concatenate	Concatenated feature maps	200×200×174
Conv2D	3×3, 1, ‘same’	200×200×196
Conv2D	3×3, 1, ‘same’	200×200×256
Conv2D	3×3, 2, ‘same’	100×100×384
Conv2D	3×3, 1, ‘same’	100×100×512
Conv2D	3×3, 2, ‘same’	50×50×640
Conv2D	3×3, 1, ‘same’	50×50×768
Conv2D_Transpose	3×3, 2, ‘same’	100×100×768
Concatenate	Skip connection	100×100×1280
Conv2D	3×3, 1, ‘same’	100×100×640
Conv2D	3×3, 1, ‘same’	100×100×512
Conv2D_Transpose	3×3, 2, ‘same’	200×200×256
Concatenate	Skip connection	200×200×512
Conv2D	3×3, 1, ‘same’	200×200×256
Conv2D	3×3, 1, ‘same’	200×200×194
Conv2D	3×3, 1, ‘same’	200×200×160
Conv2D	3×3, 1, ‘same’	200×200×128
Conv2D	3×3, 1, ‘same’	200×200×96
Conv2D	1×1, 1, ‘same’	200×200×87
AveragePool	S×S, S, ‘valid’	W×W×87

Note: W is the width of low-resolution image patches, S is the super-resolution scale. For the 2× network, S=2, W=100; for the 4× network, S=4, W=50; for the 5× network, S=5, W=40; for the 8× network, S=8, W=25; and for the 10× network, S=10, W=20.

Our purpose in this work is to generate high-resolution hyperspectral images that have spatial contrast and details as clear as the high-resolution RGB images and spectral signatures close to the original HSI. Thus, the network should be able to extract the spatial information and spectral information from the RGB images and hyperspectral images, respectively, and fuse them in the generated hyperspectral image. This was fulfilled by simultaneously minimizing the spatial loss and spectral loss of the network. For the spatial loss, both the input stacked high-resolution RGB and the generated high-resolution HSI were averaged along the spectral dimension, resulting in two high-resolution panchromatic images; then the mean squared error (MSE) of the two panchromatic images was calculated and used as the spatial loss, as Eq. (2) shows. The spectral loss calculates the MSE between the generated and input LR-HSI, as shown in Eq. (3). The loss weights of the loss functions were 0.5 and 0.5:

Lspatial=1M×N∑i=1M∑j=1N(Gi,j−G^i,j)2, (2)

Lspectral=1m×n×b∑i=1m∑j=1n∑k=1b(Hi,j,k−H^i,j,k)2  , (3)

where M and N are the spatial dimensions of the high-resolution panchromatic images; m and n are the spatial dimensions of the LR-HSI; b=87 is the number of bands in the LR-HSI; G and G^ are the average panchromatic images of the stacked HR-RGB and the generated HR-HSI, respectively; and H and $\hat{H}$ are the input and generated LR-HSI, respectively.

The unsupervised super-resolution reconstruction network was implemented using Keras on a Titan XP NVIDIA GPU with 12 GB memory. We used the Adam optimizer^37^ with a learning rate of $10^{-4}$. The network was trained with a batch size of 2. The five networks (2×, 4×, 5×, 8×, and 10×) were trained for 7 to 13 epochs depending on how fast the validation loss stopped decreasing. Each epoch took about 1 h.

### 2.4 Whole-Slide Image Classification

To evaluate the usefulness of our proposed super-resolution reconstruction network for image classification, we used the HR-RGB, original HR-HSI, and generated HR-HSI to train, validate, and test an Inception-based 2D CNN and compared the classification results. Specifically, reconstructed HR-HSI were generated for all image patches using the downsampled LR-HSI and the previously trained 4× super-resolution reconstruction network. The Inception-based CNN architecture was modified from the original Inception-v4 network^38^ to be adapted to our patch size. Each convolutional layer was initialized using the “he_normal” weight initialization^39^ and was followed by the “ReLU” activation and a 20% dropout, except the activation function of the output layer was sigmoid. The CNN architecture and the input size of each layer/block are shown in Table 3.

**Table 3:** CNN architecture for whole-slide classification.

Layer/block	Input size
Conv2D, “same”	200×200×87
Conv2D, “same”	100×100×90
Conv2D, “same”	50×50×94
Inception A block ×4	25×25×96
Reduction A block	25×25×384
Inception B block ×7	11×11×1024
Reduction B block	11×11×1024
Inception C block ×3	5×5×1536
Average pool	5×5×1536
Flatten	1×1×1536
Dense (two neurons, ‘sigmoid)	1536

The CNN was implemented using Keras^40^ with a Tensorflow backend on the same Titan XP NVIDIA GPU. The optimizer was Adadelta^41^ with an initial learning rate of 1 and decay rate of rho=0.95. The network was trained with a batch size of 16, and the loss function was binary cross-entropy. All data were split into the training, validation, and testing groups. Data from 10 patients were used for training, three patients (#3, #4, and #8) were used for validation, and three patients (#5, #14, and #15) were used for testing. No data from the same patient were used in two groups at the same time. The same data partition was used for HR-RGB, original HR-HSI, and generated HR-HSI to avoid bias. The image classification network was trained for 10 epochs, with a training time of 4.5 h per epoch using either type of hyperspectral data or 25 min per epoch using the RGB data.

### 2.5 Evaluation Metrics

To evaluate the performance of our super-resolution reconstruction network, we calculated different metrics based on the entire hypercube (spatially and spectrally), grayscale image (spatially), and spectral signatures (spectrally). We first used the MATLAB implementations of peak signal-to-noise-ratio (PSNR) and mean absolute error (MAE) to quantify the super-resolution reconstruction quality. PSNR measures the global intensity difference between generated HR-HSI and original HR-HSI and is calculated by averaging the PSNR value in a band-by-band basis across all 87 bands, with one band (B) from the real HR-HSI and the corresponding band (B^) from the generated HR-HSI:

PSNRλ(B,B^)=10 log10(peak value2/MSE),   (4)

PSNR(Real HSI, Generated HSI)=  187∑λ=187PSNRλ(B,B^), (5)

MAE was also calculated a band-by-band basis across all 87 bands:

$${\mathrm{MAE}}_{\lambda }(B,\hat{B})=\frac{1}{M\times N}\sum_{i=1}^{M}\sum_{j=1}^{N}|B_{ij}-{\hat{B}}_{ij}|,$$ (6)

MAE(Real HSI,Generated HSI)=187∑λ=187MAEλ(B,B^). (7)

For the spectral evaluation of generated HR-HSI, we used spectral angle mapper (SAM):^42^^,^^43^

$$\alpha =\cos^{-1}\;(\frac{\overset{M}{\underset{i=1}{\sum }}\overset{N}{\underset{j=1}{\sum }}t_{ij}r_{ij}}{\sqrt{\overset{M}{\underset{i=1}{\sum }}\overset{N}{\underset{j=1}{\sum }}t_{ij}^{2}\;}\sqrt{\overset{M}{\underset{i=1}{\sum }}\overset{N}{\underset{j=1}{\sum }}r_{ij}^{2}}}),$$ (8)

where $t_{ij}$ is the spectral signature from the generated HR-HSI, $r_{ij}$ is the reference spectral signature from the original HR-HSI, and α is the spectral angle between $t_{ij}$ and $r_{ij}$.

Then, we used structural similarity index measure (SSIM)^44^ and perception-based image quality evaluator (PIQUE)^45^ to evaluate the spatial character of generated HR-HSI. PIQUE was trained to evaluate the image quality without a reference image; a low score value indicates high perceptual quality. Since it only takes a grayscale or RGB image as input, we generated panchromatic images for generated HR-HSI, original HR-HSI, and stacked HR-RGB by calculating their average across all bands and then calculated PIQUE scores of the three types of panchromatic images. Usually, for super-resolution reconstruction tasks, SSIM is calculated between the entire data cube of the original and generated hyperspectral images to evaluate how “real” the generated images can be. However, due to the obvious improvement of spatial contrast in the generated HR-HSI, which resulted in a disparity between the generated and original hyperspectral images, we only obtained SSIM scores in the range of 0.3 to 0.7. Considering that most spatial information came from stacked HR-RGB, we calculated SSIM between the panchromatic images (G and G^) of stacked HR-RGB and generated HR-HSI:

$$\mathrm{SSIM}(G,\hat{G})=\frac{\left(2\mu_{G}\mu_{\hat{G}}+c_{1})(2\sigma_{G\hat{G}}+c_{2}\right)}{\left(\mu_{G}^{2}+\mu_{\hat{G}}^{2}+c_{1})(\sigma_{G}^{2}+\sigma_{\hat{G}}^{2}+c_{2}\right)},$$ (9)

where μ and σ are the mean and standard deviation of the panchromatic images, respectively, and $c_{1}={\left(0.01\times L\right)}^{2}=0.0001$ and $c_{2}={\left(0.03\times L\right)}^{2}=0.0009$ are two constants chosen as the default values dependent on the dynamic range (L) of the image values.

For the evaluation of whole-slide image classification, we use the area under the receiver operating characteristic (ROC) curve (AUC), as well as accuracy, sensitivity, and specificity as metrics. Accuracy is the ratio of all correctly labeled image patches to the total number of image patches. Sensitivity and specificity are determined by true positive (TP), true negative (TN), false positive (FP), and false negative (FN), where positive is cancerous and negative is normal. Sensitivity measures the percentage of correctly labeled cancerous image patches among all cancerous patches, and specificity measures how well normal image patches are detected:

$$\text{Accuracy}=\frac{\mathrm{TP}+\mathrm{TN}}{\mathrm{TP}+\mathrm{FP}+\mathrm{TN}+\mathrm{FN}},$$ (10)

$$\text{Sensitivity}=\frac{\mathrm{TP}}{\mathrm{TP}+\mathrm{FN}}.$$ (11)

$$\text{Specificity}=\frac{\mathrm{TN}}{\mathrm{TN}+\mathrm{FP}}.$$ (12)

## 3 Results

Our proposed unsupervised super-resolution reconstruction network fuses the spatial information from the high-resolution digital histology images and the spectral information from the low-resolution hyperspectral images and generates high-quality, high-resolution hyperspectral images. The reconstruction time for each 200×200  pixels image patch was about 100 ms. The quantitative evaluation results of five super-resolution reconstruction networks (2×, 4×, 5×, 8×, and 10×) are shown in Table 4. The PSNR, SSIM, and PIQUE measures are more related to the spatial component of the hyperspectral images, whereas the MAE and SAM are more sensitive to the spectral reconstruction errors.^46^ The PSNR and MAE of five networks indicate a satisfying reconstruction performance both spatially and spectrally. Due to the significant improvement in image quality of the generated HR-HSI, especially the recovered texture details that were missing in the original HR-HSI, we did not get high SSIM values on the entire hyperspectral data cube. However, we calculated SSIM between the panchromatic images of generated HR-HSI and stacked HR-RGB and got an average SSIM of 93.1% (2×), 95.0% (4×), 96.0% (5×), 96.5% (8×), and 97.0% (10×) in the validation data, as well as 89.6% (2×), 93.2% (4×), 94.6% (5×), 95.9% (8×), and 96.7% (10×) in the testing data, which showed high-level similarity of spatial components in generated HR-HSI and HR-RGB. Considering the inherent difference between the two panchromatic images, which would have lowered the SSIM value to a certain extent, the obtained SSIM scores indicate good maintenance of the spatial information. The PIQUE scores for the panchromatic images of generated HR-HSI were comparable to the stacked HR-RGB (28.1 for validation and 30.0 for testing) and were lower than the original HR-HSI (32.5 for validation and 37.5 for testing), also proving a significantly improved image quality of the generated hyperspectral data. Regarding the spectral signatures, the average spectral angles of all networks were lower than 5 deg, showing good maintenance of spectral information. Still, due to the improved image contrast of the generated HR-HSI, the spectral signature of each pixel in generated HR-HSI is not always the same as that of the corresponding pixel in the original HR-HSI. It can be noticed that the network with a larger scale usually results in a higher SSIM score and larger spectral angle because there is less spectral information from the input, and the output is prone to rely more on the spatial information from HR-RGB.

**Table 4:** Quantitative validation and testing results of the unsupervised super-resolution reconstruction network.

	Scale	PSNR (dB)	MAE (%)	SAM (deg)	SSIM (%)	PIQUE
Validation	2×	25.1	4.4±0.6	2.9	93.1	26.6
	4×	24.3	4.8±0.9	3.5	95.0	25.1
	5×	23.2	5.5±0.9	3.8	96.0	26.6
	8×	22.7	5.8±0.8	4.2	96.5	27.4
	10×	22.3	6.1±0.9	4.2	97.0	28.6
Testing	2×	23.8	5.0±1.1	2.9	89.6	27.6
	4×	22.8	5.7±1.5	3.7	93.2	25.4
	5×	21.6	6.4±1.7	3.9	94.6	27.5
	8×	21.1	7.1±1.7	4.4	95.9	29.8
	10×	20.7	7.4±2.0	4.5	96.7	30.9

**Fig. 6:**
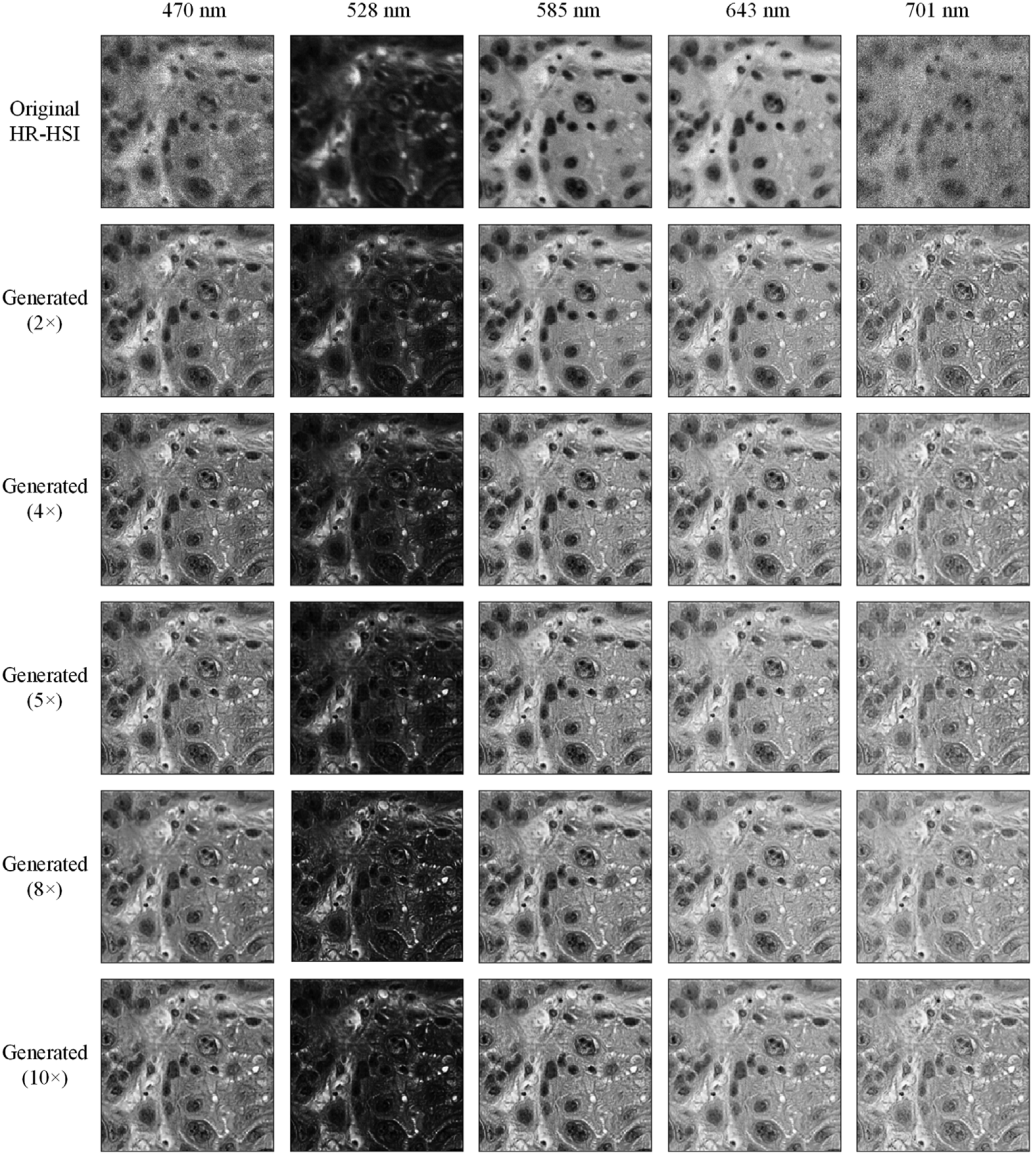
Single-band images of the original HR-HSI and generated HR-HSI from five different super-resolution reconstruction networks showing high similarity and satisfying spatial reconstruction in various wavelength bands. The major difference is in the first (e.g., 470 nm) and last (e.g., 701 nm) several bands, where noise exists in the original HSI but gets removed from the generated HSI.

**Fig. 7:**
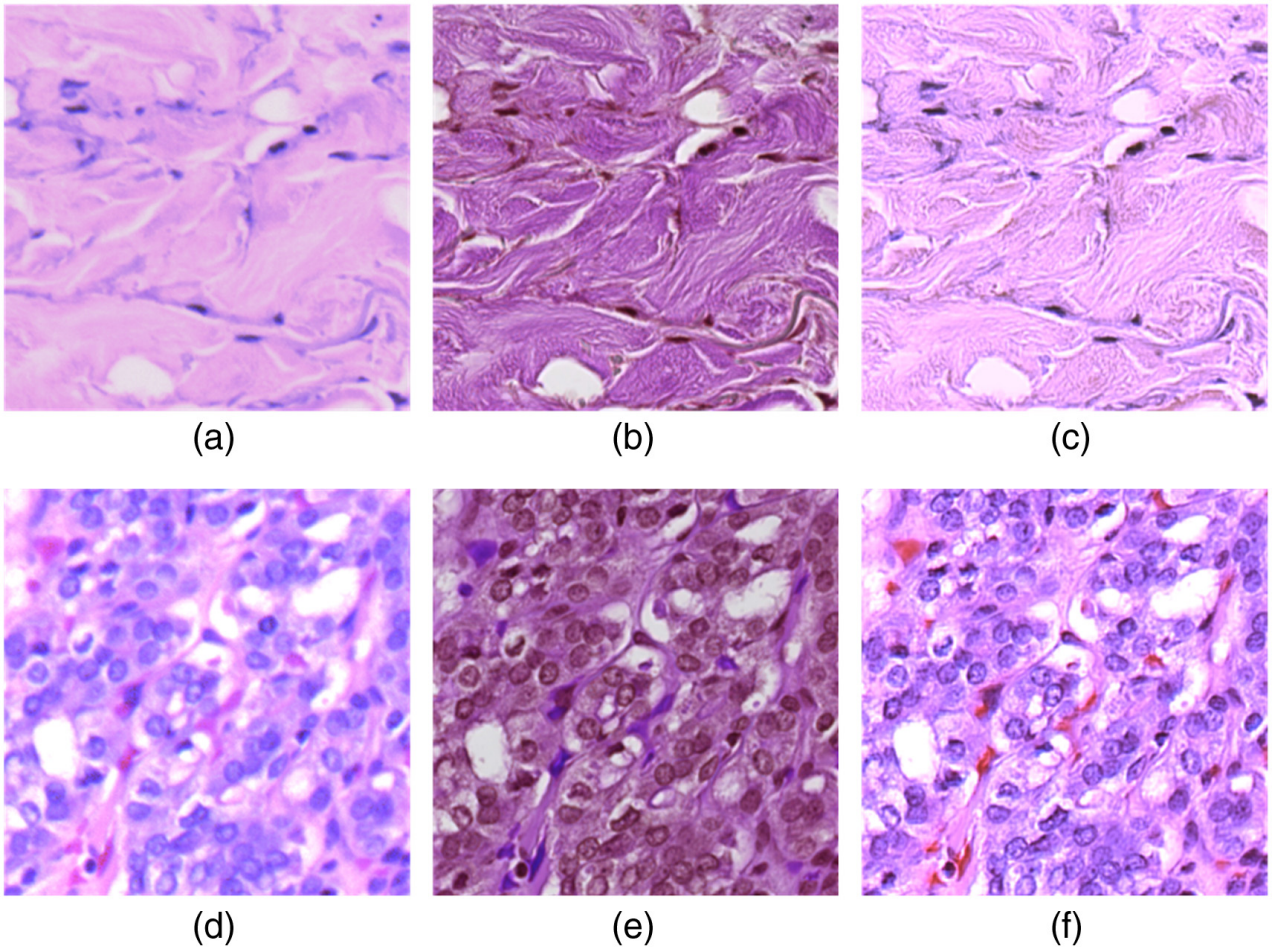
Improvement of image quality in generated high-resolution hyperspectral images. (a) HSI-synthesized RGB of an original high-resolution hyperspectral image patch, which was out-of-focus and lost the fiber structures due to an accidental impromptu focus adjustment. (b) Coregistered high-resolution RGB histology image patch with fine structures of the tissue. (c) HSI-synthesized RGB of the generated high-resolution hyperspectral image patch, where the fiber structures are recovered. (d) HSI-synthesized RGB patch from a partially out-of-focus image, where the edges of nuclei become fuzzy. (e) Coregistered high-resolution RGB histology image with clear edges of nuclei. (f) HSI-synthesized RGB of the generated HR-HSI, where the shape of nuclei is well recovered.

Figure 6 shows five wavelength bands of the original HR-HSI and generated HR-HSI from different super-resolution reconstruction networks. The intensity of each wavelength band in the generated HR-HSI is close to that in the original HR-HSI. The spatial features were very well reconstructed in all wavelength bands, even if some texture details were missing in the original HR-HSI (e.g., 701 nm). It can be seen that the RGB-guided super-resolution reconstruction network significantly reduced the noise in some wavelength bands (e.g., 470 nm) that was caused by the low sensitivity of the image sensor. Moreover, our network also compensates for the unsatisfying focusing issue on some hyperspectral images. The high-frequency spatial information that was fused from the RGB images also increased the contrast in the generated hyperspectral images, especially when the hyperspectral camera was not perfectly focused and the image quality was compromised, as shown in Fig. 7. Specifically, Fig. 7(a) shows an HSI-synthesized RGB image patch that was out-of-focus due to an accidental impromptu focus adjustment, and Fig. 7(d) is from a partially out-of-focus image caused by uneven slide thickness. Our network was able to extract the spatial information from the digital histology images, as shown Figs. 7(b) and 7(e), and recover the fiber structures as well as clear nuclei edges in the reconstructed images, as shown in Figs. 7(c) and 7(f). Note that the out-of-focus images in this figure are just examples to illustrate the performance of our proposed method, and they do not imply intrinsic low image quality of HSI.

**Fig. 8:**
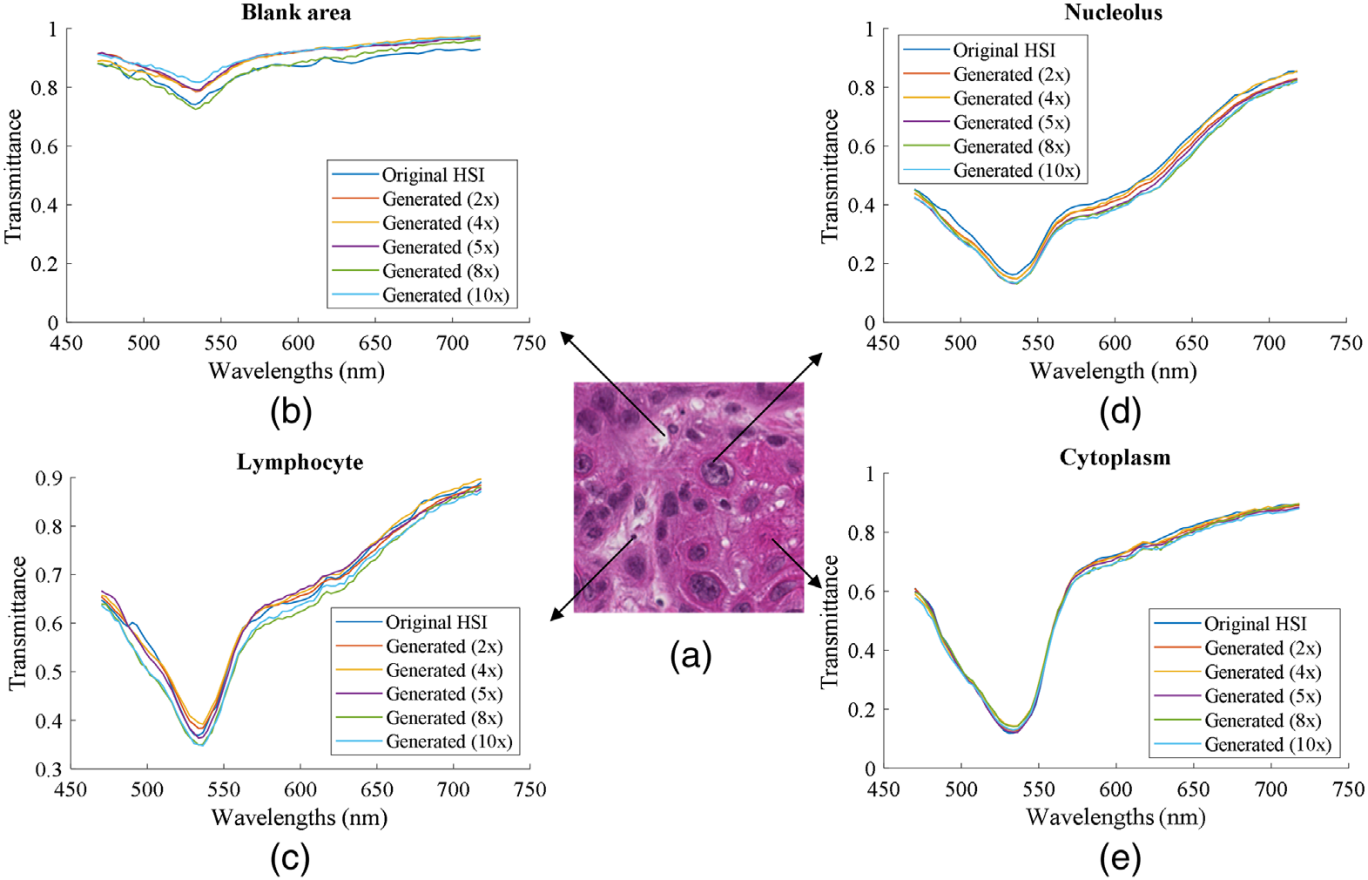
Comparison of spectral signatures of various cellular components from the original HR-HSI and generated HR-HSI. The well-maintained spectral shapes indicate a good spectral reconstruction. (a) A high-resolution digital histology image patch from a head and neck cancer slide, including cancer cells and lymphocytes. (b) Spectral signatures of the same blank area extracted from the original HR-HSI and the generated HR-HSI. (c) Spectral signatures of the same lymphocyte extracted from the original HR-HSI and the generated HR-HSI. (d) Spectral signatures of the same nucelous from a cancer cell, extracted from the original HR-HSI and the generated HR-HSI. (e) Spectral signatures of cytoplasm extracted from the same region in the original HR-HSI and the generated HR-HSI.

Figure 8 shows the spectral signatures of the extracted nucleus, cytoplasm, lymphocyte, and blank area in the slide from the original HR-HSI and the generated HR-HSI, respectively. The shape of spectra from different tissue types was well maintained, which is critical for effective image classification.

**Fig. 9:**
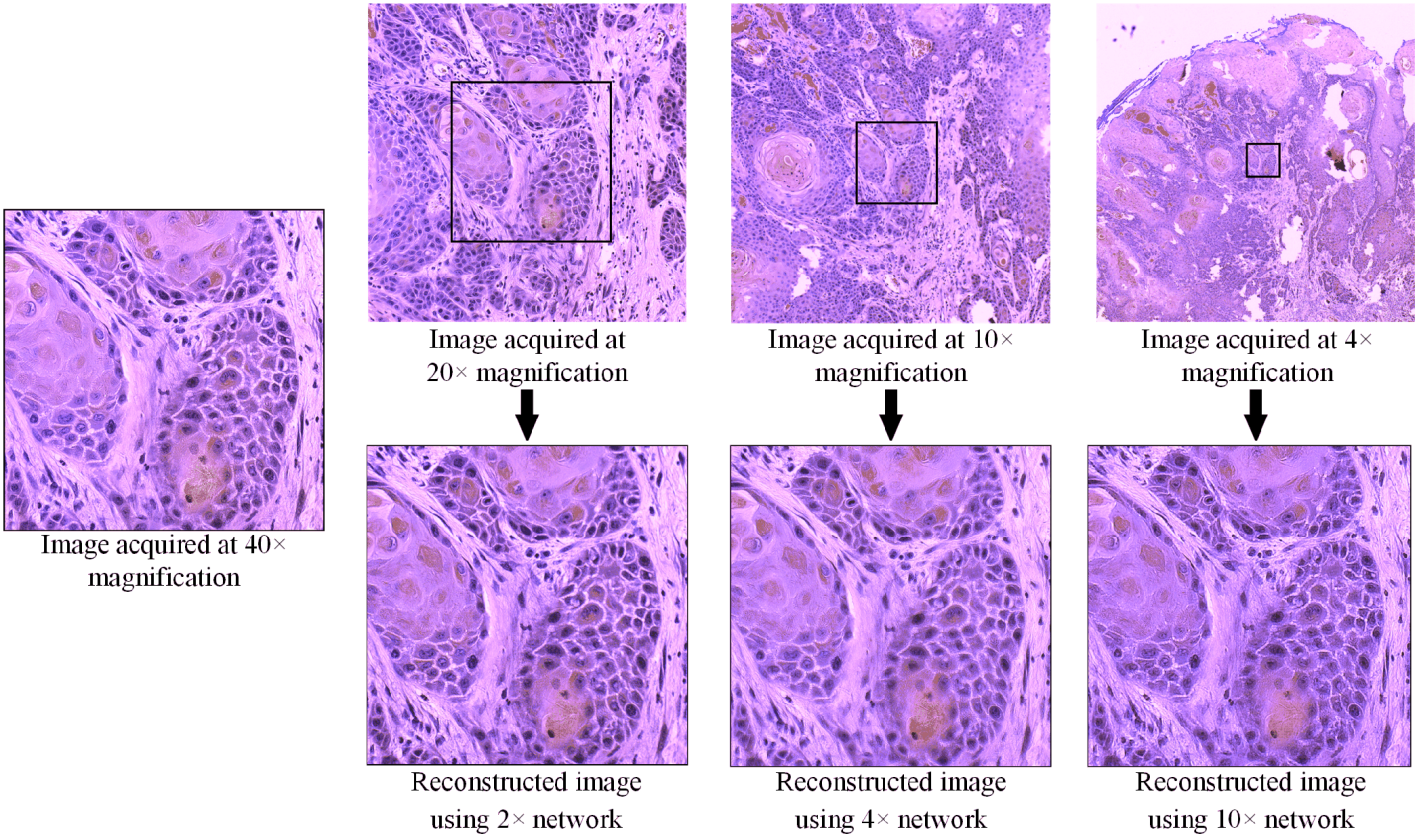
Super-resolution reconstruction using images with physically different resolutions. The black contours in the low-resolution images outline the same region as the high-resolution image acquired at 40× magnification.

To further validate the effectiveness of our proposed super-resolution reconstruction network, we acquired hyperspectral images of the same region on a histologic slide using four objective lenses with different magnifications, namely 4×, 10×, 20×, and 40×. Then, we reconstructed high-resolution hyperspectral images (equivalent to the optical resolution with the 40× objective lens) using the three relatively low-resolution images, i.e., the images acquired at 20×, 10×, and 4× objective magnification. Figure 9 shows the synthesized RGB images of the hyperspectral images acquired with physically different optical resolutions as well as the reconstructed images. The black square contours in the low-resolution images show the same region of interest as the high-resolution image. It can be seen that the detailed structures were very well recovered in the reconstructed images, and the significant similarity of colors in the synthesized images indicates a satisfying maintenance of the spectral characteristics. Therefore, despite our network being trained using generated low-resolution hyperspectral data, it works well on real low-resolution images.

In addition to the abovementioned evaluation metrics, we implemented image classification using the original HR-HSI and generated HR-HSI to further prove the usefulness of our proposed super-resolution reconstruction network. The validation and testing results using three types of data are shown in Table 5. RGB histology images, which contained morphological and color information of the slides, had satisfying classification results of 0.9 AUC, 0.85 accuracy, 0.88 sensitivity, and 0.77 specificity in the validation group, as well as 0.86 AUC, 0.80 accuracy, 0.82 sensitivity, and 0.76 specificity in the testing data group. The high image resolution, together with the autofocusing and other image enhancement techniques integrated with the whole-slide scanner, guaranteed sufficient spatial details of the anatomical structures, which were critical for the classificantion. The prediction time using either hyperspectral data or RGB data was <30 ms per patch. For the classification of a whole slide, the prediction time varied from 0.5 to 3 min with the size of the slide.

**Table 5:** Quantitative results of whole-slide image classification using different data.

	Data	AUC	Accuracy	Sensitivity	Specificity
Validation	HR-RGB	0.90	0.85	0.88	0.77
	Original HR-HSI	0.93	0.87	0.89	0.87
	Generated HR-HSI	0.94	0.90	0.89	0.90
Testing	HR-RGB	0.86	0.80	0.82	0.76
	Original HR-HSI	0.87	0.82	0.85	0.73
	Generated HR-HSI	0.90	0.86	0.87	0.83

Although the hyperspectral camera was focused by our manual adjustment, which might be subjective and dependent on the operators, it was able to acquire hyperspectral images with a very decent image quality. Regardless of the noise in a few wavelength bands and some partially unfocused images, which would have slightly compromised the spatial characters, the original high-resolution hyperspectral image patches still outperformed RGB because of the extra spectral information, with 0.93 AUC, 0.87 accuracy, 0.89 sensitivity, and 0.87 specificity in validation data, as well as 0.87 AUC, 0.82 accuracy, 0.85 sensitivity, and 0.73 specificity in the testing group. As for the generated HR-HSI, the unsupervised super-resolution reconstruction network fused the spatial components from the RGB images and the spectral information from the hyperspectral images, resulted in an improved image quality in the generated HR-HSI. One significant improvement was the removal of noise, which greatly increased the efficacy of the first and last several bands. In addition, the out-of-focus images, which were caused by the uneven thickness of slides and had fuzzy nuclei edges or blurry fiber structures, also regained sharpness. Due to the improved image quality, using generated HR-HSI made the classification performance even better.

**Fig. 10:**
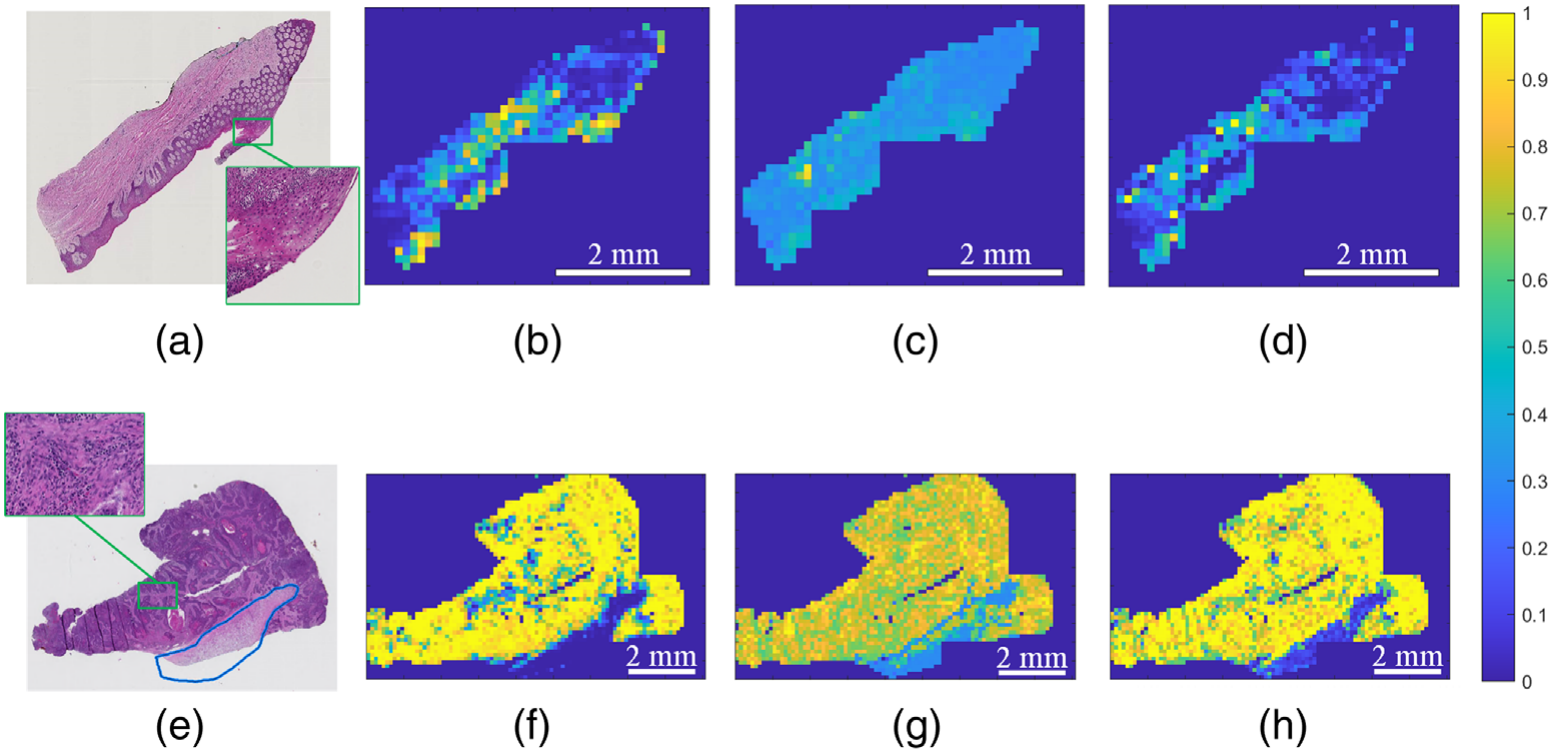
Illustration of whole-slide image classification using different types of data. (a) The digital histology image of a normal tissue slide. (b)–(d) Probability map of the normal slide using the RGB image patches, original hyperspectral image patches, and generated hyperspectral image patches, respectively. Note that the green contour in (a) was falsely classified as positive in (b) using RGB image patches but was correctly classified as negative using either hyperspectral image patches (c and d). (e) The digital histology image of a tumor slide with a little dysplasia tissue (regarded as normal) at the bottom. The green rectangle region was falsely classified as negative using RGB image patches but was correctly classified using hyperspectral patches. (f)–(h) Probability maps of the tumor slide using RGB image patches, original hyperspectral image patches, and generated hyperspectral image patches, respectively.

Figure 10 shows the probability maps of whole-slide classification using the HR-RGB, original HR-HSI, and generated HR-HSI. Although using RGB image patches achieved satisfying results, it is prone to generate false positives at deformed epithelium or false negatives where very few cancer nuclei exist, as shown in Figs. 10(a)–10(b), 10(e)–10(f). Classification using the original hyperspectral image patches obviously reduced the number of false positives and false negatives, which proved the usefulness of the spectral information. However, the probability values of tumor and normal images using the original HR-HSI were not as extremely separated. Instead, most tumor patches had a probability value of around 0.8, and most normal patches had a probability value of 0.2 to 0.3. This does not affect the classification results though, and it is likely to be improved by increasing the dataset. The probability maps of generated HR-HSI show improved classification performance compared with both HR-RGB and original HR-HSI. It seems that the high-contrast spatial components from the high-resolution RGB images might have provided critical cancer-related information, such as the morphology of cancerous nuclei,^33^ and the spectral components from the hyperspectral images were beneficial especially where RGB images tend to give false results. In addition, at the bottom of the tumor slide in Fig. 10(e), there is a small piece of tissue with dysplasia, which is a type of abnormalty but not tumor. None of the networks misclassified it without being previously trained, although the probability maps of HR-HSI did show abnormalty to some extent.

## 4 Discussion and Conclusion

In this work, we developed an unsupervised CNN based on a modified U-Net architecture for hyperspectral super-resolution reconstruction with the guidance of high-quality RGB digital histology images. The network fuses the spatial information from the high-resolution RGB images and the spectral information from the low-resolution hyperspectral images to generate high-resolution hyperspectral images. The generated hyperspectral images from all five super-resolution reconstruction networks with different spatial enhancement scales (2×, 4×, 5×, 8×, and 10×) maintained the shape of the original spectral signatures while being enriched with high-frequency spatial information. In addition, the proposed method improved the image quality for hyperspectral images, including reducing image noise, increasing image contrast, and deblurring the image. Furthermore, the unsupervised method does not require any high-resolution hyperspectral image as ground truth for network training, which minimizes the complexity of the workflow. We implemented image classification using the RGB image patches, original hyperspectral image patches, and hyperspectral patches generated from the 4× network, respectively. The results show the usefulness of the spectral information in the hyperspectral images and prove the ability of our proposed super-resolution reconstruction network for improving image quality. By fusing the high-contrast spatial information from the RGB histology images and the rich spectral information from the hyperspectral images, our proposed method potentially brings benefits to histopathological diagnosis.

One shortcoming of this study is the small dataset volume. To avoid potential data leakage between the super-resolution reconstruction network and the whole-slide image classification network, we only used a very small portion of data (two images per slide) for the super-resolution reconstruction network and left the remainder for the whole-slide image classification network. It is possible that, with more training data, our super-resolution reconstruction network could perform even better for spectral signature reconstructions. As for the whole-slide image classification network, 32 entire slides may not be sufficient due to the anatomical variations of SCC and the significantly increased number of features in the hyperspectral data. In addition, the tissues in all slides were either tumor or normal, which made it slightly easier to get decent image classification results. In the future, we will investigate whole-slide cancer detection in tumor-normal margin slides and see if the generated HR-HSI is able to significantly improve the outcome.

Another shortcoming in this work is that we used the simulated low-resolution hyperspectral data to train the super-resolution reconstruction network, instead of acquiring a large number of hyperspectral images with a physically lower resolution as training data. It is possible that the differences between the real low-resolution hyperspectral images and simulated ones may affect the network outcome. But acquiring images with different optical resolutions and registering the high-resolution RGB images to the low-resolution hyperspectral images potentially introduces misalignment and thus minor errors during the network training. On the other hand, the way that we simulated the LR-HSI for training data (downsampling with “box” kernel) was the same as how the network output the generated LR-HSI, which could have reduced some bias. In addition, we tested the network trained with simulated LR-HSI on real LR-HSI to validate the performance of the proposed method. The results indicate that, even though the network was trained with simulated low-resolution data, it also worked well with images with actual lower resolutions. In the future, we will further investigate this by acquiring more images with physically different resolutions.

With HSI getting more attention in the medical imaging field, the conflict among the acquisition speed, data storage, and resolution of hyperspectral images must be solved to apply this technology in real clinical settings. Our proposed method makes it possible to generate high-quality hyperspectral images for automatic histopathological analysis with a low-resolution HSI camera and low-magnification objective lens, hence greatly reducing the acquisition time and the file size of hyperspectral histologic images. For instance, with a 4× network, the acquisition time of a line-scanning HSI system can be cut to 1/4, and the file size can be cut to 1/16. It also allows for the use of snapshot cameras, which can easily reach video-rate imaging, without the concern of low spatial image resolution. Moreover, our method does not change the routine workflow in pathology. Instead of developing a new complex system, it can be achieved simply by mounting a low-resolution hyperspectral camera onto a commercial whole-slide scanner. The acquired low-resolution hyperspectral images can be stored and used for automatic digital pathological analysis after super-resolution reconstruction, while the RGB digital histology images are still available for pathologists to peruse and confirm the results.

In the future, we plan to develop an automatic whole-slide scanning HSI microscope with a low-resolution hyperspectral camera (e.g., a snapshot camera) and the proposed super-resolution reconstruction network built into the system. By synchronizing the color camera and the hyperspectral camera, the system will be able to acquire both data simultaneously. With the mature autofocusing and image enhancement techniques of color cameras as well as our proposed method, the image quality of both the RGB and hyperspectral images will be secured. We may be able to establish a comprehensive database of whole-slide hyperspectral histologic images, which is extremely beneficial for a thorough investigation of pathological features in hyperspectral images as well as various deep learning algorithms. In addition, the super-resolution reconstruction network and whole-slide image classification network may be combined into a single and straightforward workflow in which high-resolution hyperspectral images can be generated along with the acquisition of low-resolution hyperspectral images and high-resolution RGB images and directly fed into the image classification network, so that histopathological diagnosis can be achieved during the scanning of the slides.

In conclusion, our deep learning-based hyperspectral image super-resolution reconstruction method can bring high-resolution spatial features and rich spectral information to improve pathological diagnosis, while increasing the image acquisition speed and reducing data storage requirement, which will provide wide applications in digital and computational pathology.
