# Unsupervised super-resolution reconstruction of hyperspectral histology images for whole-slide imaging (Errata)

**DOI:** 10.1117/1.JBO.27.5.059801

**Published:** 2022-05-23

**Authors:** Ling Ma, Armand Rathgeb, Hasan Mubarak, Minh Tran, Baowei Fei

**Affiliations:** a University of Texas at Dallas, Department of Bioengineering, Richardson, Texas, United States; b Tianjin University, State Key Laboratory of Precision Measurement Technology and Instruments, Tianjin, China; c The University of Texas at Dallas, Center for Imaging and Surgical Innovation, Richardson, Texas, United States; d The University of Texas Southwestern Medical Center, Department of Radiology, Dallas, Texas, United States

## Abstract

Corrections to the published article include values in Table 1 and provision of an omitted reference.

This article [*J. Biomed. Opt.* **27**(5), 056502 (2022) doi: 10.1117/1.JBO.27.5.056502 was originally published on 16 May 2022 with the following errors:

1. The wavelength range in Section 2.1 was misstated as 480 to 720 nm; the correct wavelength range is 470 to 720 nm.
2. Values in Table 1 representing the numbers of patches in the hyperspectral histologic dataset were incorrectly reported as follows:

Original:

**Table 1:** Summary of the hyperspectral histologic dataset.

Patient ID	Organ	Number of images			Number of patches		
		T slide	N slide	Total	Cancerous	Normal	Total
1	Larynx	58	42	100	16,667	9756	26,423
2	Hypopharynx	95	45	140	21,040	17,350	38,390
3	Larynx	38	62	100	10,077	9563	19,640
4	Larynx	22	28	50	3949	8171	12,120
5	Larynx	58	23	81	14,526	5508	20,034
6	Larynx	29	10	39	6903	2139	9042
7	Larynx	28	16	44	7087	2992	10,079
8	Buccal mucosa	23	12	35	4888	1841	6729
9	Larynx	20	37	57	3979	8199	12,178
10	Larynx	13	19	32	3111	4604	7715
11	Larynx	53	24	77	13,573	3671	17,244
12	Larynx	23	17	40	5375	2694	8069
13	Larynx	58	28	86	16,318	6401	22,719
14	Larynx	46	28	74	12,263	6518	18,781
15	Larynx	35	17	52	8750	4238	12,988
16	Larynx	81	40	121	20,002	10,073	30,075
**Total**		680	448	1128	168,508	103,718	272,226

Corrected:

**Table:** 

Patient ID	Organ	Number of images			Number of patches		
		T slide	N slide	Total	Cancerous	Normal	Total
1	Larynx	58	42	100	16,444	9756	26,200
2	Hypopharynx	95	45	140	21,040	17,350	38,390
3	Larynx	38	62	100	11,077	16,924	28,001
4	Larynx	22	28	50	5015	6819	11,834
5	Larynx	58	23	81	14,526	5508	20,034
6	Larynx	29	10	39	6903	2139	9042
7	Larynx	28	16	44	7087	2992	10,079
8	Buccal mucosa	23	12	35	4888	1841	6729
9	Larynx	20	37	57	3979	8199	12,178
10	Larynx	13	19	32	3111	4604	7715
11	Larynx	53	24	77	13,573	3671	17,244
12	Larynx	23	17	40	5375	2694	8069
13	Larynx	58	28	86	16,318	6401	22,719
14	Larynx	46	28	74	12,519	6518	19,037
15	Larynx	35	17	52	8750	4238	12,988
16	Larynx	81	40	121	20,002	10,073	30,075
**Total**		680	448	1128	170,607	109,727	280,334

3. A reference was omitted: K. He et al., “Delving deep into rectifiers: surpassing human-level performance on ImageNet Classification,” Proc. IEEE International Conference on Computer Vision, 1026-1034, (2015). The reference was added as Ref. 39; Refs. 39-45 were changed to Refs. 40-46.

These errors were corrected on 18 May 2022.

